# Acute Lung Injury, Repair, and Remodeling: Pulmonary Endothelial and Epithelial Biology

**DOI:** 10.1155/2017/9081521

**Published:** 2017-03-14

**Authors:** Yutong Zhao, Karen Ridge, Jing Zhao

**Affiliations:** ^1^Department of Medicine, University of Pittsburgh, 3459 Fifth Avenue, 628 NW MUH, Pittsburgh, PA 15213, USA; ^2^Department of Medicine, Northwestern University, McGaw Pavilion Suite M-410, 240 E Huron, Chicago, IL 60611, USA

Acute lung injury (ALI) and ARDS are defined by damage to the alveolar epithelium and endothelium, which allows the exudation of protein-rich fluid into the alveolar space. ALI/ARDS are life threatening inflammatory lung diseases, most commonly caused by local or systemic inflammation, including pneumonia and sepsis. An uncontrolled cytokine storm leads to detrimental effects such as cell death, epithelial and endothelial barrier disruption, and edema. Lung repair and remodeling following ALI are crucial steps and could be a risk factor for determining morbidity and mortality. During normal repair and remodeling phases, alveolar fluid is reabsorbed and debris from dead cells and pathogens is cleared by mononuclear-linage phagocytes. Additionally, endothelial cells regenerate, epithelial cells proliferate and differentiate, and collagen fibers are formed at site of injury. Finally, excess deposits of collagen fibers are processed and removed.

Pulmonary edema is a major complication of ALI. Approaches aiming at improving and maintaining endothelial and epithelial barrier integrity and function are in high demand. The aim of this special issue is to illuminate pulmonary endothelium and epithelium biological function during ALI, repair, and remodeling. We have selected 6 original research manuscripts and 1 review article covering the range from basic research aspects to translational studies and therapeutic evaluation.

ALI/ARDS is associated with a high mortality rate between 20 and 50% and is one of the main clinical complications in severe malaria. M. L. M. Pereira et al. determine the protective role of heme oxygenase-1 (HO-1) and HO-1 inducing drug, hemin, in malaria-associated ALI. This study will potentially advance therapeutic approach in prevention of ALI/ARDS development in severe malaria. In attempt to manage the uncontrolled cytokine storm induced, a NEMO-binding domain peptide (NBD) was developed by J. Huang et al. to inhibit LPS-induced NF-*κ*B activation; NBD limits cytokine release, neutrophil infiltration, and pulmonary vascular leakage in LPS-induced ALI mouse model. As an analysis of the enhancement of endothelium barrier integrity aspect, L. Wang et al. have investigated the protective role of pan-caspase inhibitor Q-VD in cytomix-induced pulmonary microvascular endothelial cell permeability and apoptosis. In the review article, Y. Zhuang et al. have addressed microRNA regulation in endothelial junction proteins and clinical consequence.

Lipid mediators, prostaglandin E2 (PGE_2_) and Leukotriene B_4_ (LTB_4_), play an important role in host defense in many infections. The study by L. C. Rodrigues et al. has unveiled that Gal-1 protects against histoplasmosis by maintaining the balance of nitric oxide (NO) and PGE2. AM966 is an antagonist of lysophosphatidic acid receptor 1, which potentiates antifibrotic function in experimental study. The study by J. Cai et al. has revealed that AM966 treatment induces lung microvascular endothelial barrier disruption which is regulated by RhoA/MLC and phosphorylation of VE-cadherin. Further, P. Geraghty et al. demonstrate the ER stress in cigarette smoke-induced COPD.

In conclusion, many factors contribute to the pathogenesis of ALI/ARDS. It is important to understand the pathophysiological mechanisms of ALI/ARDS to improve the outcome of ALI patients. These special research articles were selected to offer readers updated knowledge on new therapeutic approach development for ALI/ARDS and precaution in clinical trial medicine.

*Yutong Zhao* *Karen Ridge* *Jing Zhao*

